# Wedelolactone Enhances Osteoblastogenesis but Inhibits Osteoclastogenesis through Sema3A/NRP1/PlexinA1 Pathway

**DOI:** 10.3389/fphar.2016.00375

**Published:** 2016-10-18

**Authors:** Yan-Qiu Liu, Xiao-Fei Han, Jun-Xia Bo, Hui-Peng Ma

**Affiliations:** ^1^Institute (College) of Integrative Medicine, Dalian Medical UniversityDalian, China; ^2^Glucose and Lipid Metabolism Laboratory of Liaoning Province, College of Life Science and Technology, Dalian UniversityDalian, China; ^3^College of Medical Laboratory, Dalian Medical UniversityDalian, China

**Keywords:** osteoblastogenesis, osteoclastogenesis, wedelolactone, Sema3A, NRP1

## Abstract

Bone remodeling balance is maintained by tight coupling of osteoblast-mediated bone formation and osteoclast-mediated bone resorption. Thus, agents with the capacity to regulate osteoblastogenesis and osteoclastogenesis have been investigated for therapy of bone-related diseases such as osteoporosis. In this study, we found that wedelolactone, a compound isolated from *Ecliptae herba*, and a 9-day incubation fraction of conditioned media obtained from wedelolactone-treated bone marrow mesenchymal stem cell (BMSC) significantly inhibited tartrate-resistant acid phosphatase (TRAP) activity in RANKL-stimulated osteoclastic RAW264.7 cells. Addition of the semaphorin 3A (Sema3A) antibody to the conditioned media partially blocked the medium’s inhibitory effects on the RAW264.7 cells. In BMSC, mRNA expression of Sema3A increased in the presence of different wedelolactone concentrations. Blocking Sema3A activity with its antibody reversed wedelolactone-induced alkaline phosphatase activity in BMSC and concurrently enhanced wedelolactone-reduced TRAP activity in osteoclastic RAW264.7 cells. Moreover, in BMSC, wedelolactone enhanced binding of Sema3A with cell-surface receptors, including neuropilin (NRP)1 and plexinA1. Furthermore, nuclear accumulation of β-catenin, a transcription factor acting downstream of wedelolactone-induced Sema3A signaling, was blocked by the Sema3A antibody. In osteoclastic RAW264.7 cells, conditioned media and wedelolactone promoted the formation of plexin A1-NRP1, but conditioned media also caused the sequestration of the plexin A1-DNAX-activating protein 12 (DAP12) complex and suppressed the phosphorylation of phospholipase C (PLC)γ2. These data suggest that wedelolactone promoted osteoblastogenesis through production of Sema3A, thus inducing the formation of a Sema3A-plexinA1-Nrp1 complex and β-catenin activation. In osteoclastic RAW264.7 cells, wedelolactone inhibited osteoclastogenesis through sequestration of the plexinA1-DAP12 complex, induced the formation of plexinA1-Nrp1 complex, and suppressed PLCγ2 activation.

## Introduction

Bone homeostasis is maintained by osteoclast-mediated bone destruction and osteoblast-mediated bone formation, which are two tightly coupled and controlled processes. An imbalance between both bone resorption and formation can result in metabolic bone diseases such as osteoporosis (Martin and Sims, 2005). Therefore, agents for regulation of the balancing mechanisms are important for osteoporosis therapy. Many coupling factors have been identified for regulating the coordination of osteoblastogenesis and osteoclastogenesis. For example, differentiation and maturation of osteoclasts (derived from monocyte/macrophage precursor cells) can be regulated by osteoblasts, which express key osteoclast differentiation factors such as receptor activator of nuclear factor kappa-B ligand (RANKL; Teitelbaum and Ross, 2003). In addition, to counterbalance the activity of these stimulatory coupling factors, osteoblasts can produce inhibitory coupling factors, including osteoprotegerin, sclerostin, bone morphogenetic protein 6 (BMP6), and Wnt10b (Balemans et al., 2001; Brunkow et al., 2001; Bennett et al., 2005, 2007).

Sema3A is a member of the semaphorin family, a group of proteins involved in the development of the nervous, immune systems and bone (Negishi-Koga and Takayanagi, 2012; Worzfeld and Offermanns, 2014). Sema3A, produced by osteoblasts, has been identified as a potent and direct inhibitor of osteoclast formation from osteoclast precursor cells (Gomez et al., 2005; Hayashi et al., 2012). Distinct from other coupling factors, Sema3A promotes osteoblast differentiation from bone marrow mesenchymal stem cells (the precursor of osteoblasts), indicating a dual function role in which it inhibits osteoclastogenesis and enhances osteoblastogenesis (Fukuda et al., 2013). The Sema3A signaling pathway is activated through binding with its cell-surface receptor composed of an Nrp1 and plexinA1 protein complex, which functions as a signal-transducing subunit (Gu et al., 2003; Worzfeld and Offermanns, 2014). This complex induces different downstream signaling molecules in osteoclasts and osteoblasts, resulting in different regulatory effects on differentiation. Therefore, regulation of the Sema3A pathway in osteoclasts and osteoblasts would be promising for the bone remodeling balance and be helpful for the development of therapeutic agents.

Wedelolactone is a small molecular compound isolated from *Ecliptae herba.* In China, *Ecliptae herba* is used as an herbal kidney-nourishing drug and also is commonly believed to have the ability to strengthen bones. It is also used to treat bone diseases such as osteoporosis. Wedelolactone has been reported to possess various biological activities, including inhibition of 5-lipoxygenase and typsin, antagonizing myotoxins, and inducing caspase-dependent apoptosis (Wagner and Fessler, 1986; Melo and Ownby, 1999; Syed et al., 2003; Sarveswaran et al., 2012). Recently, it was reported that *Ecliptae herba* extract showed a therapeutic effect on bone metabolism of ovariectomized rats (Zhang et al., 2013). We previously reported that wedelolactone, as the major active constituent in *Ecliptae herba*, inhibited the proliferation and differentiation of osteoclastic RAW264.7 and mouse monocytes from blood (Liu et al., 2014).

In this study, we used wedelolactone-treated bone marrow mesenchymal stem cell (BMSC)-derived conditioned medium to treat osteoclastic RAW264.7 cells. The differentiation and bone resorptive activity of RAW264.7 cells was evaluated. Interestingly, compared to RANKL-treated alone, RAW264.7 cells exposed to conditioned media for 4 days showed a significant decrease in osteoclastic differentiation and function. The production of Sema3A from BMSC was evaluated. The role of wedelolactone-induced Sema3A/Plexin A1/NRP1 pathway in BMSC and in osteoclastic RAW264.7 cells was then subsequently investigated.

## Materials and Methods

### Isolation and Culture of Mouse Bone Marrow Mesenchymal Stem Cells (BMSCs)

BMSC were isolated according to a previously published protocol with some modifications (Krebsbach et al., 1999; Zhang et al., 2009). Briefly, BMSC were isolated from bone marrow, which was aspirated from 8-week old BALB/c mice. All procedures involving mice were carried out in compliance with the relevant laws and institutional guidelines, and approved by the Ethics Committee at Dalian Medical University. BMSC were collected using gradient centrifugation of mesenchymal stem cell-specific gradient solutions (Tianjin Haoyang Biological Manufacture, China). A layer of the bone marrow cell fraction in phosphate-buffered saline (PBS) was placed on top of the gradient solution and centrifuged at 340 *g* for 20 min. The cell fraction was collected and washed with PBS after centrifugation. The cell samples were resuspended in Minimum Essential Medium Alpha Medium (α-MEM, Gibco, Paisley, UK), supplemented with 10% fetal calf serum (FCS), 100 U/ml penicillin, and 100 μg/ml streptomycin, and maintained at 37°C with 5% CO_2_ in a humidified atmosphere. On day 3, the cell suspension was replaced with fresh complete medium. BMSC were further separated from hematopoietic cells by their differential adhesion to tissue culture plastic and prolonged proliferation potential. After 6–7 days in culture, 90% confluence was reached. These cell samples were used in the experiment.

### Conditioned Media Preparation

BMSC were cultured with osteogenic medium (OS) (100 nM dexamethasone, 1 mM β-glycerophosphate, and 5 μM L-ascorbic acid 2-phosphate) + 2 μg/ml wedelolactone. Wedelolactone was provided by Key Laboratory of Separation Science for Analytical Chemistry at Dalian Institute of Chemical Physics, Chinese Academy of Sciences (Dalian, China). The purity was >98%. These cells were incubated for 12 days at 37°C with 5% CO_2_ in a humidified incubator. Every 3 days the medium was changed and collected. The 3-, 6-, 9-, and 12-day incubation fractions containing wedelolactone-treated or -untreated conditioned media were harvested for use.

### Culture of Pre-osteoclastic RAW264.7 Cells

Mouse pre-osteoclastic RAW264.7 cells were purchased from the Type Culture Collection of Chinese Academy of Sciences (Shanghai, China). The cells were cultured in DMEM (Gibco, Grand Island, NY, USA) supplemented with 10% FCS, 0.03% L-glutamine (Gibco), penicillin (100 U/ml), and streptomycin (100 μg/ml) and maintained at 37°C with 5% CO_2_ in a humidified atmosphere.

For differentiation, cells were plated in DMEM or conditioned medium supplemented with 30 ng/ml recombinant RANKL. For drug assays, wedelolactone was added to the culture medium at different concentrations. Cells were incubated for 4 days at 37°C with 5% CO_2_ in a humidified incubator and fed daily with RANKL-supplemented medium.

### MTT Assay

RAW264.7 cells were plated at a density of 1 × 10^4^ cells per well of 96-well plates. After overnight incubation, RANKL, wedelolactone, or conditioned media was added to the plates. Following incubation for 6 days, cell growth was measured by 3-(4, 5-dimethylthiazol2-yl)-2,5-diphenyltetrazolium bromide (MTT) with a plate reader (Tecan, Switzerland) as previously described (Liu et al., 2014). The percentage of proliferation was calculated with the formula:

Proliferation rate (%)⁢=A492(sample)/A492(control)⁢×100%.

### Measurement of TRAP Activity

RAW264.7 cells were fixed with 60% citrate buffered acetone for 30 s. The fixed cells were then washed with water 3× and were further incubated with 100 μl phosphatase substrate solution containing 10 mM pNPP and 10 mM sodium tartrate in 50 mM citrate buffer (PH 4.6) at 37°C for 1 h. After incubation, the enzyme reaction mixture was transferred to another plate, and the reaction was stopped with 100 μl of 0.1 N NaOH. Absorbance at 405 nm was measured using an ELISA reader (Tecan, Switzerland).

For staining of tartrate-resistant acid phosphatase (TRAP), cells were fixed with 60% citrate buffered acetone for 30 s. The cells were then stained for TRAP with 0.1 M acetate solution (PH 5.0) containing 6.76 mM sodium tartrate, 0.12 mg/ml naphthol AS-MX phosphate, and 0.07 mg/ml of fast Garnet GBC solution as described in the manufacturer’s instruction (Sigma, St. Louis, MO, USA). Photomicrographs were obtained using an Olympus microscope at 200× magnification.

### ALP Activity Assay

For *in vitro* osteoblast differentiation, mouse BMSC were isolated from 8-week old BALB/c mice according to a previously published protocol, and were cultured with α-MEM with 10% FBS. After 5 days, cells were reseeded (1 × 10^4^ per cm^2^) and cultured with OS. Culture medium was changed every third day. After 9 days, alkaline phosphatase (ALP) staining (Sigma) and activity measurements were performed by using an ALP staining kit according to manufactory’s instruction.

### Western Blot Analysis

For Western blot analysis, cells were lysed using lysis buffer containing 10 mM Tris/HCl (PH 7.5), 150 mM NaCl, 2 mM EDTA, 1% (v/v) Triton X-100, 1 mM Na_3_CO_4_, 1 mM PMSF, and 0.1 mM aprotinin. The cells were scraped from the plates and centrifuged at 16,060 *g* for 30 min at 4°C. For nuclear protein extraction, the pellet from the 700 *g* centrifugation was washed by buffer A, resuspended in buffer B containing 20 mM HEPES, pH 7.9, 1.5 mM MgCl_2_, 420 mM NaCl, 0.2 mM EDTA, 10 mM NaF, 2 mM Na_3_VO_4_, 1 mM pyrophosphoric acid, and Complete TM protease inhibitors (Cell Signaling, USA) and incubated on ice for 5 min. The protein concentration in the cell lysates was determined using the Bradford protein assay. Western blot analysis was performed as previously described using the several antibodies (Bennett et al., 2005). Sema3A, plexin A1, and NRP1 antibodies were obtained from ABcam, Cambridge, UK. PLCγ2, phospho-PLCγ2, β-cateinin, and DAP12 antibodies were from Cell Signaling, USA.

### Immunoprecipitation Assay

For immunoprecipitation assay, cells were harvested and lysed in radioimmuno- precipitation assay (RIPA) buffer. Cell lysates were then centrifuged at 12,000 rpm for 10 min at 4°C (Hayashi et al., 2012). PlexinA1 or IgG antibodies were added into the supernatant and incubated at 4°C overnight with rotation. Protein A or G agarose beads (Thermo, Rockford, IL, USA) were then added and incubated with rotation for 3 h at 4°C. After centrifugation, proteins were subjected to Western blotting.

### Quantitative Real-Time RT-PCR

All work was carried out in a designated PCR-clean area. RNA was extracted from cells using Trizol reagent (Gibco-BRL, Rockville, MD, USA) and isolated as specified by the manufacturer. The RNA was DNAse-treated (DNase I-RNase-Free, Ambion) to remove any contaminating DNA; 200 ng of total RNA was reverse-transcribed with oligodT primers using the High Capacity cDNA RT Kit (Takara, Japan) in a 20-μl cDNA reaction, as specified by the manufacturer. For quantitative PCR, the template cDNA was added to a 20 μl reaction with SYBR GREEN PCR Master Mix (Applied Biosystems) and 0.2 μM of primer. The amplification was carried out using an ABI Prism 7000 for 40 cycles under the following conditions: (1) an initial denaturation of 95°C for 10 min plus 40 cycles of 95°C for 15 s and (2) 60°C for 1 min. The -fold changes were calculated relative to β-actin (NM_007393.5) using the ΔΔCt method for Sema3A (NM_001243073.1) mRNA analysis. The following primer sets consisted of mouse β-actin: forward, 5′-GTACGCCAACACAGTGCTG-3′; reverse, 5′-CGTCATACTCCTGCTTGCTG-3′ and mouse Sema3A: forward, 5′-AGTGTCCGTACGATCCCAAG-3′; reverse, 5′-GAACAGTGACCACCGTCATC-3′.

### Statistical Analysis

Differences between experimental groups were evaluated by one-way analysis of variance (ANOVA) using SPSS 17.0 software. Differences with a *P*-value < 0.05 were considered statistically significant. All experimental data are presented as the mean ± SEM with values from more than three experiments.

## Results

### Effect of Conditioned Media from Wedelolactone-Treated BMSC on Osteoclastic RAW264.7 Differentiation

**FIGURE 1:**
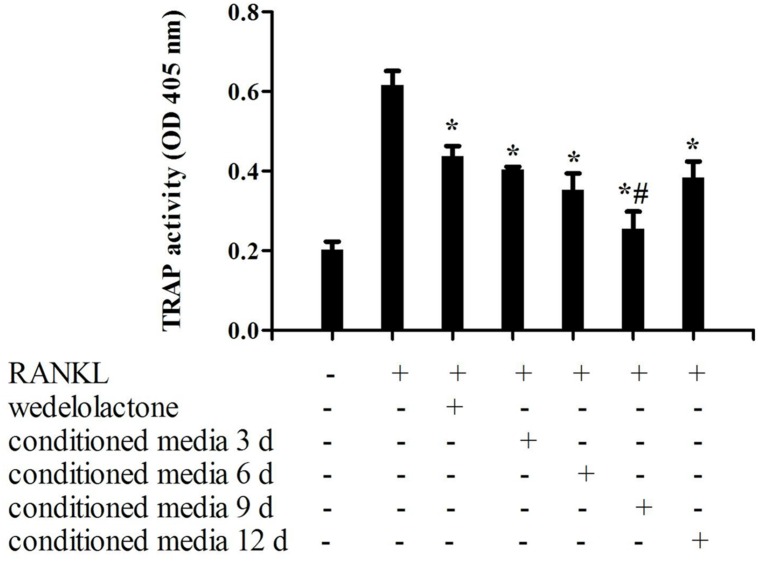
**Effect of wedelolactone-treated bone marrow mesenchymal stem cell (BMSC) conditioned media on osteoclastic differentiation of RAW264.7 cells.** Different fractions of wedelolactone-treated BMSC conditioned media were added to RAW264.7 cells. After 4 days of incubation, cultures were fixed, and TRAP activity was assayed. Data are expressed as mean ± SEM of three independent experiments. ^∗^*P* < 0.05 compared with RANKL-treated cells. ^#^*P* < 0.05 compared with wedelolactone-treated cells.

To determine the influence of wedelolactone-treated conditioned media from BMSC on osteoclastic differentiation, TRAP activity of osteoclastic RAW264.7 cells was examined after treatment with wedelolactone or conditioned media. As shown in **Figure 1**, when RAW264.7 cells were exposed to RANKL for 4 days, TRAP activity was significantly increased. After addition of different conditioned media fractions, which were treated with 2 μg/ml wedelolactone for 3, 6, 9, and 12 days, TRAP activity decreased but to different levels. The decreased amount of TRAP activity was most potent with the addition of the 9-day conditioned medium fraction (**Figure 1**). The effects of wedelolactone and 9-day conditioned medium on RAW264.7 cell viability was evaluated.

**FIGURE 2:**
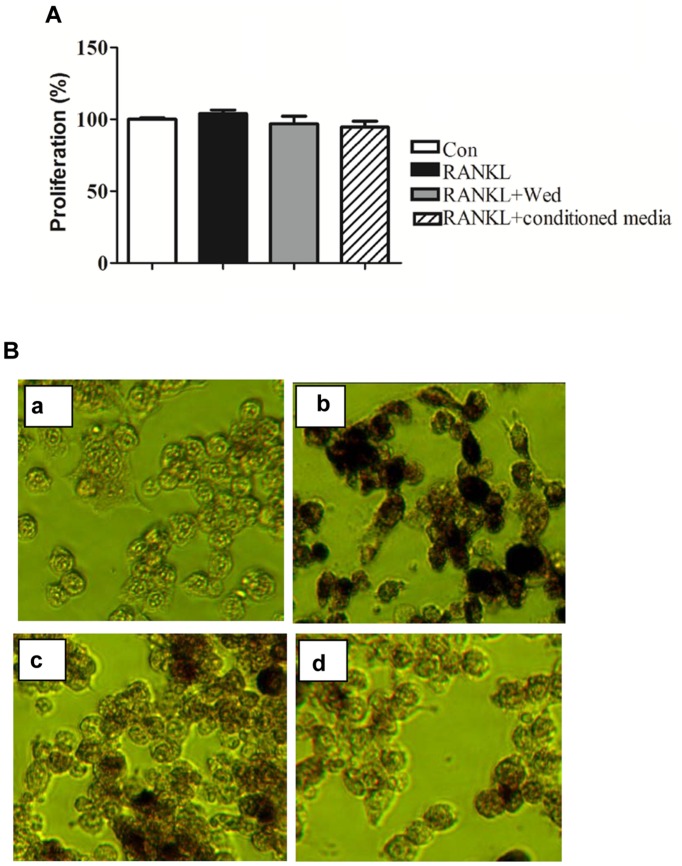
**Effect of wedelolactone-treated BMSC conditioned media on RAW264.7 cell proliferation and TRAP activity. (A)** RAW264.7 cells were treated with RANKL, wedelolactone or a 9-day conditioned medium fraction for 4 days. An MTT assay was then performed; *n* = 3, mean ± SEM. **(B)** RAW264.7 cells were incubated with the 9-day conditioned medium fraction for 4 days. RAW264.7 cells were treated with conditions as follows: (a) control; (b) RANKL; (c) RANKL+ wedelolactone; (d) RANKL+ conditioned media for 9-day fraction. Cells were then stained for TRAP by using a TRAP staining kit and imaged at 200×.

The 9-day conditioned medium fraction or wedelolactone at 2 μg/ml did not influence cell viability in the MTT assay (**Figure 2A**). However, TRAP staining confirmed that the RANKL-induced increased number of TRAP-stained positive RAW264.7 cells was significantly reduced by incubation with 2 μg/ml wedelolactone. In contrast, treatment with the 9-day conditioned medium fraction led to a more significant decrease in the number of TRAP-stained positive RAW264.7 cells (**Figure 2B**).

### Effect of Wedelolactone on the Production of Sema3A from BMSC

**FIGURE 3:**
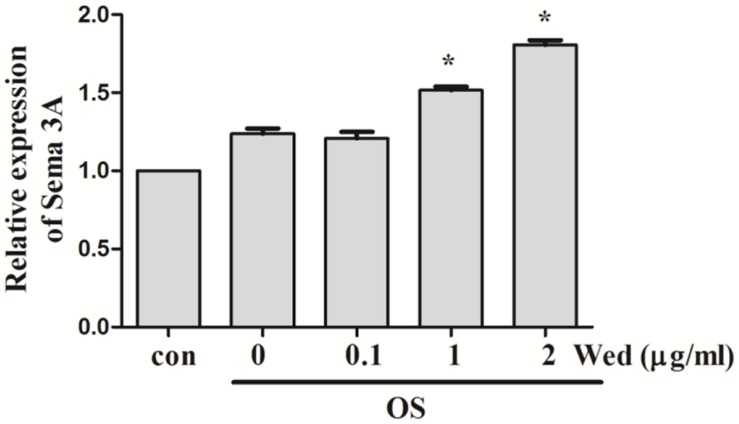
**Effect of wedelolactone on Sema3A mRNA expression in BMSC.** BMSC were incubated with OS medium containing wedelolactone for 9 days. Total RNA was then isolated. Gene expression was analyzed by rt-qPCR with Sema3A specific primer sets. The expression levels were normalized to the housekeeping genes Actb. Data are expressed as mean ± SEM of three independent experiments. ^∗^*P* < 0.05 compared with OS-treated control.

**FIGURE 4:**
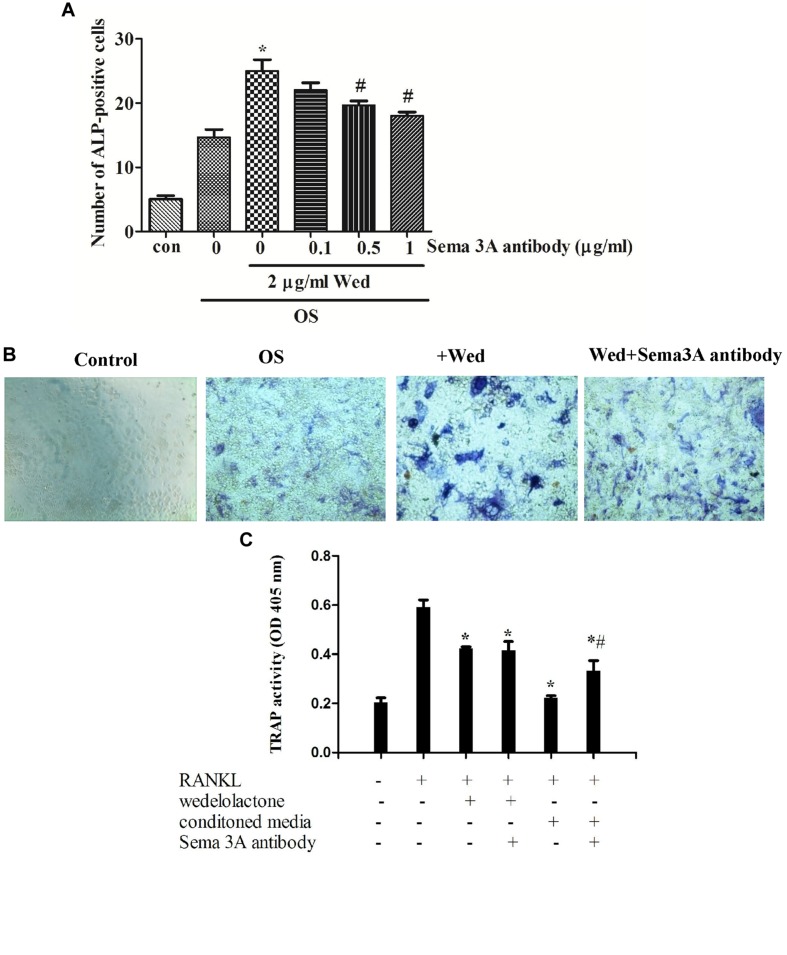
**Effects of Sema3A antibody on wedelolactone-induced ALP activity in BMSC and TRAP activity in RAW264.7 cells. (A)** BMSC were cultured in OS medium containing 2 μg/ml wedelolactone and Sema3A antibody for 9 days. The cells were fixed and stained for ALP activity. The data represent the mean ± SEM (*n* = 3). **(B)** ALP staining for BMSC. The cells were treated with conditions as follows: (a) control; (b) OS; (c) OS+wedelolactone; (d) OS+wedelolactone + Sema3A antibody for 4 days. Cells were then stained for ALP by using a ALP staining kit and imaged at 200×. **(C)** TRAP activity in RAW264.7 cells were assayed after the cells were treated with conditioned media for 9-day fraction or Sema3A antibody for 4 days. The data represent the mean ± SEM (*n* = 3). ^∗^*P* < 0.05 compared with RANKL-treated cells. ^#^*P* < 0.05 compared with conditioned media-treated cells.

Sema3A is reported to be produced from osteoblasts (Hayashi et al., 2012). To examine whether wedelolactone stimulated the production of Sema3A from BMSC, qRT-PCR assay was first performed. It was observed that the Sema3A mRNA expression level was upregulated after wedelolactone treatment of BMSC for 9 days (**Figure 3**). We added different concentrations of Sema3A antibody to BMSC, and then the activity of ALP, a marker enzyme for osteoblast differentiation, was determined. The increased number of ALP staining-positive BMSC by wedelolactone was reduced by addition of 0.5 or 1 μg/ml Sema3A antibody (**Figure 4A**). Morphological changes further confirmed that 1 μg/ml Sema3A antibody blocked the increase in the number of ALP-staining positive BMSC (**Figure 4B**). In addition, although 1 μg/ml Sema3A antibody did not affect the reduction in wedelolactone-induced TRAP activity in osteoclastic RAW264.7 cells, the decrease in TRAP activity caused by exposure to the 9-day conditioned medium fraction was reversed by Sema3A antibody (**Figure 4C**). These results suggest that wedelolactone-stimulated Sema3A production contributed to the enhancement of ALP activity in BMSC and inhibition of TRAP activity in RAW264.7 cells.

### Wedelolactone Regulates Osteoblastogenesis through Sema3A/NRP1/PlexinA1 Pathway-Mediated β-Catenin Activation

**FIGURE 5:**
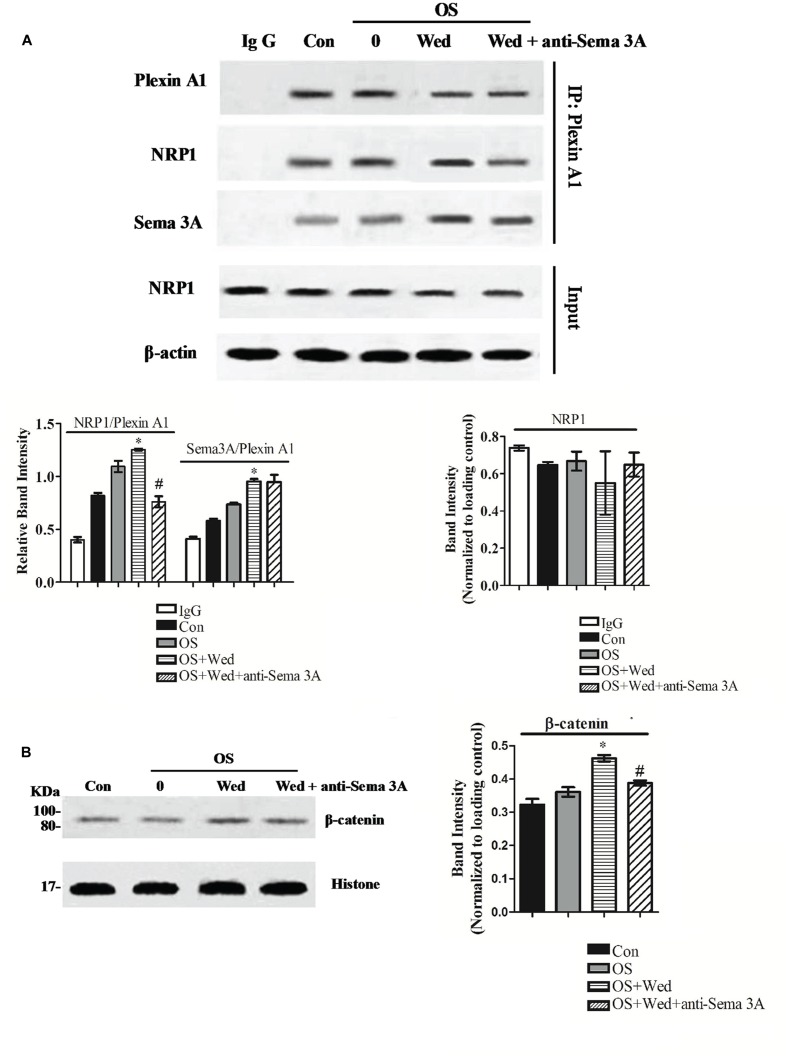
**Effect of wedelolactone on the formation of Sema3A/NRP1/PlexinA1 complex and nuclear accumulation of β-catenin in BMSC.** BMSC were seeded in 10-cm plate and treated with or without 2 μg/ml wedelolactone for 9 days. The cells were lysed and subjected to coimmunoprecipitation assays **(A)** or Western blot assays **(B)**. Data are expressed as mean ± SEM of three independent experiments. ^∗^*P* < 0.05 compared with OS-treated control. ^#^*P* < 0.05 compared with wedelolactone-treated cells.

The activity of Sema3A on osteoblasts is mediated by its binding to the cell surface receptor consisting of Nrp1 and plexinA1. To determine the mechanism by which wedelolactone stimulated the production of Sema3A and subsequently enhanced osteoblastogenesis, the binding of NRP1, plexin-A1, and DAP12 was examined after treatment of BMSC with wedelolactone. Wedelolactone treatment (2 μg/ml) enhanced the binding of NRP1 and plexin-A1, while inhibition of Sema3A by its antibody reduced the formation amount of NRP1-plexin-A1 complex. The formation of the Sema3A-plexin-A1 complex was increased by wedelolactone, but the Sema3A antibody had no effect on the increased amount of the wedelolactone-induced Sema3A-plexin-A1 complex (**Figure 5A**).

The Sema3A signaling pathway is known to induce activation of the Wnt/β-catenin signaling pathway, which is crucial for osteoblast differentiation (Hens et al., 2005; Pederson et al., 2009). Therefore, we focused on nuclear translocation of β-catenin, a transcription factor involved in osteoblast differentiation. When BMSC were treated with wedelolactone, an increase in nuclear accumulation of β-catenin was observed. However, addition of the Sema3A antibody reversed the enhanced wedelolactone-induced nuclear translocation (**Figure 5B**). These results suggest that wedelolactone promoted the formation of a Sema3A-NRP1-plexin-A1 complex and subsequently activated β-catenin, which resulted in BMSC differentiation toward osteoblasts.

### Involvement of Plexin A1/Nrp1/DAP12 Pathway Activation during Inhibition of Osteoclastogenesis by Wedelolactone

**FIGURE 6:**
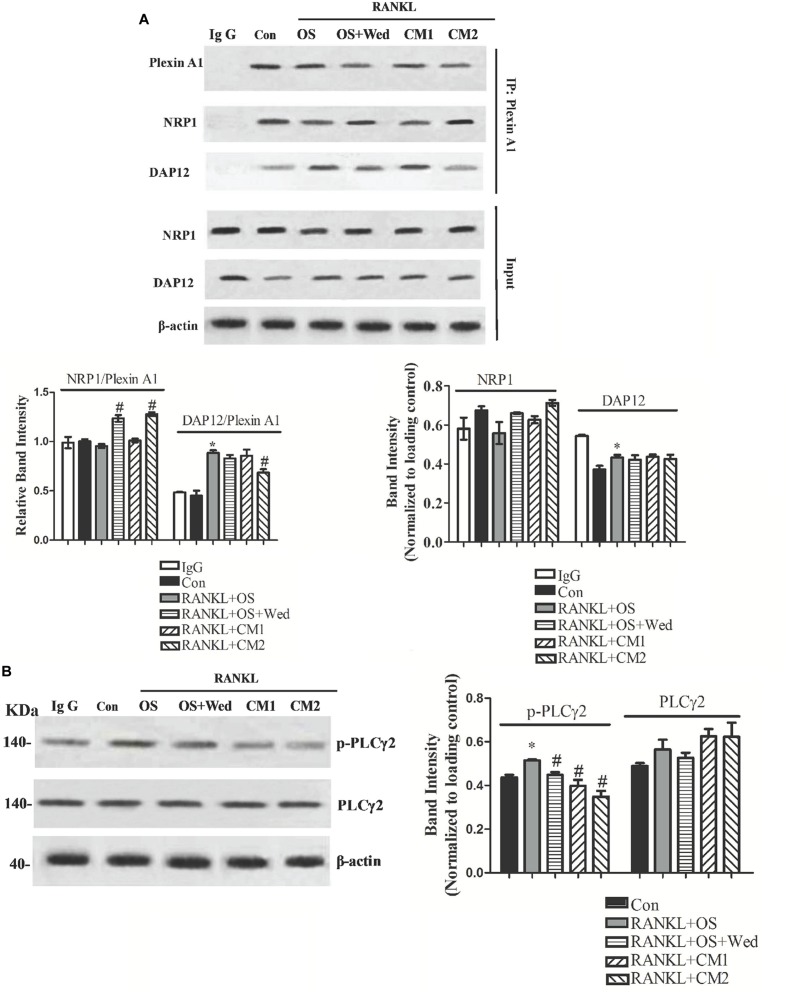
**Effect of wedelolactone on the formation of NRP1-PlexinA1 and PlexinA1-DAP12 complex, and PLCγ2 activation in RAW264.7 cells.** RAW264.7 cells were treated with RANKL for 4 days in the presence of OS, wedelolactone, BMSC conditioned media (CM1) and wedelolactone-treated conditioned media for 9-day fraction (CM2). Then the cells were lysed and subjected to coimmunoprecipitation assays **(A)** or Western blot assays **(B)**. The data represent the mean ± SEM (*n* = 3). ^∗^*P* < 0.05 compared with untreated control. ^#^*P* < 0.05 compared with RANKL+OS group.

In contrast to osteoblasts, the activity of Sema3A on osteoclasts is mediated through inhibition of RANKL-induced formation of the plexinA1-DAP12 complex but without affecting the plexinA1-Nrp1 complex. To examine the impact of wedelolactone on the activation of the Sema3A signaling pathway in osteoclasts, the expression of plexinA1 associated with Nrp1 or DAP12 was determined. As shown in **Figure 6A**, addition of RANKL to RAW264.7 cells induced the formation of the plexinA1-DAP12 complex by sequestering plexinA1 from Nrp1, which was consistent with that of previously reported results (Hayashi et al., 2012). The RANKL-induced increase in the amount of DAP12 associated with plexinA1 was downregulated by conditioned media, while the decrease in the amount of Nrp1 associated with PlexinA1 was upregulated by wedelolactone. Furthermore, incubation with the 9-day conditioned medium fraction enhanced the effect of wedelolactone on the plexinA1-NRP1 complex formation, indicating that wedelolactone combined with Sema3A activated the plexinA1-NRP1 pathway. Phospholipase C (PLC)γ2 acts downstream of the plexinA1/NRP1/DAP12 pathway, which participates in osteoclastogenesis. To determine whether PLCγ2 activation was involved in wedelolactone-reduced osteoclastogenesis, phosphorylation of PLCγ2 was monitored. RANKL-induced phosphorylation of PLCγ2 was inhibited by wedelolactone treatment. Addition of the 9-day conditioned medium fraction resulted in a more significant decrease in phosphorylation of PLCγ2 expression (**Figure 6B**), indicating that wedelolactone activated the plexin A1//NRP1/DAP12 pathway and subsequently inhibited PLCγ2 activation, thus leading to reduced osteoclastogenesis.

## Discussion

Bone homeostasis requires the coupling of bone resorption and bone formation. An excess of osteoclastic bone resorption over osteoblastic bone formation can result in bone-related diseases such as osteoporosis. Osteoblasts and osteoclasts are coupled by various coupling factors, which maintain bone remodeling homeostasis. Some coupling factors such as sclerostin, which is produced from osteoclastic precursors, reduce bone formation. Some osteoclast-derived coupling factors such as sphingosine 1-phosphate, however, induced osteoblast differentiation (Goetzl et al., 2000). Interestingly, Sema3A, as a dual function coupling factor, which is secreted by osteoblastic cells, inhibits osteoclastic activity and at the same time, promotes osteoblastic differentiation (Hayashi et al., 2012). It appears to be useful for preventing bone loss. Mice lacking Sema3A were found to have undergone bone loss (Fukuda et al., 2013). Therefore, regulation of Sema3A and its corresponding signaling pathway may be helpful for treatment of osteoporosis (Behar et al., 1996). In cultured chick dorsal root ganglion neurons, several small molecular drugs have been reported to interfere with Sema3A activity through inhibition of Sema3A-induced growth cone collapse (Kikuchi et al., 2003; Kaneko et al., 2006). However, it has not been shown that small molecular compounds stimulate Sema3A activity to prevent of bone loss. In this study, wedelolactone, a compound isolated from *Ecliptae herba*, stimulated Sema3A mRNA expression, indicating that the wedelolactone’s mechanism of action appeared to be promotion of Sema3A activity, distinct from the known Sema3A activity inhibitor.

Wedelolactone is a component isolated from *Ecliptae herba*, which is widely used in China to strengthen bones. Our previous study showed that wedelolactone inhibited RANKL-induced osteoclast differentiation (Liu et al., 2014). In this study, we further investigated the role of wedelolactone in the coupling of osteoclast and osteoblasts during earlier precursor stages. Mature osteoblastic cells are derived from BMSC, which secret various factors, including Wnt and BMP, to induce differentiation toward osteoblasts (Jaiswal et al., 1997; Hens et al., 2005). Upon 9 days of treatment with wedelolactone, the mRNA expression levels of Sema3A increased. A 9-day conditioned medium fraction was further confirmed to contain biologically active Sema3A protein, as confirmed by inhibition of ALP activity by Sema3A antibody. However, fractions obtained from more or less than 9 days of incubation (such as 6 or 12 days) had no obvious inhibitory effect on TRAP activity of RAW264.7 cells, suggesting that Sema3A derived from BMSC was induced by wedelolactone at the 9-day differentiation stage. These results were similar to those from a previous study of upregulated Sema3A mRNA expression from calvarial cells stimulated by 1α, 25 (OH)_2_D_3_ and PGE_2_ (Hayashi et al., 2012).

In osteoblasts, Sema3A was shown to associate with plexin A1 and Nrp1, which induces the downstream activation of the Wnt pathway (Narazaki and Tosato, 2006). Sema3A production from wedelolactone-stimulated BMSC resulted in the formation of the plexin A1-Nrp1 and plexin A1-Sema3A complexes, indicating that wedelolactone-induced osteoblast differentiation was mediated through the Sema3A/Plexin A1/Nrp1 pathway. Blockage of active Sema3A by addition of Sema3A antibody reversed the increase in the amount of the plexin A1-Nrp1 complex but had no effect on the amount of the plexin A1-Sema3A complex, indicating that addition of Sema3A antibody was not enough to block the binding of plexin A1 with Sema3A; wedelolactone, however, was able to promote the formation of the plexin A1-Sema3A and plexin A1-Nrp1 complexes. β-Catenin has been shown to be a key transcription factor of Wnt pathway, which is responsible for osteoblastogenesis (Day et al., 2005). A previous study reported that Wnt3a-induced nuclear accumulation of β-catenin was suppressed in Sema3A^-/-^ calvarial cells (Hayashi et al., 2012). In this study, wedelolactone treatment resulted in the nuclear accumulation of β-catenin. However, neutralization of Sema3A by addition of the Sema3A antibody partially inhibited nuclear accumulation of β-catenin, indicating that wedelolactone-stimulated β-catenin activation through nuclear translocation acted downstream of the plexin A1-Nrp1 complex.

In osteoclasts, RANKL has been reported to downregulate Nrp1 expression, resulting in osteoclast differentiation via sequestration of plexin A1 from Nrp1 with formation of plexin A1-DAP12 complex (Takahashi and Strittmatter, 2001; Takegahara et al., 2006). In this study, the RANKL-induced formation of the plexin A1-DAP12 complex in osteoclastic RAW264.7 cells was inhibited by conditioned media and wedelolactone, while the amount of plexin A1 associated with Nrp1 increased. This result was consistent with the previous study that reported Sema3A binding to the plexin A1-Nrp1 complex followed by suppression of RANKL-induced osteoclast differentiation via a reduction in the formation of the plexin A1-DAP12 complex (Hayashi et al., 2012). Addition of Sema3A antibody reversed the effects of conditioned media on the formation of the plexin A1-Nrp1 complex and PLCγ2 activation, since PLCγ2 phosphorylation was indicated to be related to Sema3A signaling (Mao et al., 2006; Epple et al., 2008; Faccio and Cremasco, 2010). In the current study, in addition to the role of Sema3A in the conditioned media, wedelolactone was also implicated in direct mediation of the Sema3A/plexin A1/Nrp1 pathway instead of the plexin A1/DAP12 pathway. However, we do not know whether the action of wedelolactone did or did not directly interfere with the interaction of the Sema3A/plexin A1/Nrp1 pathway. Additional studies concerning the target for wedelolactone on osteoblasts and osteoclasts are needed. A previous study showed that wedelolactone inhibited breast cancer-induced osteoclastogenesis through inhibition of IκBα phosphoration (Hsieh et al., 2015). NF-κB pathway acts downstream of Sema3A signaling. The upregulated expression of Nrp1 by wedelolactone might be attributed to inhibition of the NF-κB pathway.

Of note, quite a few studies indicate that plexins and semphorins family proteins have multiple roles. Genetic as well as pharmacological *in vivo* experiments have indicated that therapeutic interventions with Sema3A may also cause adverse effects (Goshima et al., 2012). Wedelolactone is suggested to regulate osteoblasts and osteoclasts by stimulating Sema3A activity. Concurrently, wedelolactone was able to alter other pathways such as the NF-κB pathway. This multi-target effect of wedelolactone might prevent the side effects of Sema3A.

## Conclusion

In the present study, we demonstrated that effective stimulation of Sema3A production from BMSC by wedelolactone contributed to osteogenesis. Wedelolactone and wedelolactone-treated 9-day conditioned medium fraction from BMSC exerted inhibitory effects on osteoclastogenesis via Sema3A/plexin A1/Nrp1 signaling.

In BMSC, wedelolactone induced the binding of plexin A1/Nrp1 and activated downstream molecules of Wnt/β-catenin pathway.

In osteoclastic RAW264.7 cells, the plexin A1-DAP12 complex was sequestered by wedelolactone with subsequent inhibition of PLCγ2 phosphorylation.

## Author Contributions

Y-QL, X-FH, and H-PM participated in research design, performed data analyses, and contributed to writing, revising, and final approval of the manuscript. Y-QL and J-XB conducted the experiments.

## Conflict of Interest Statement

The authors declare that the research was conducted in the absence of any commercial or financial relationships that could be construed as a potential conflict of interest.

## References

[B1] Balemans W. M. Ebeling N. Patel E. Van Hul P. Olson M. Dioszegi M. (2001). Increased bone density in sclerosteosis is due to the deficiency of a novel secreted protein (SOST). *Hum. Mol. Genet.* 10 537–543. 10.1093/hmg/10.5.537 11181578

[B2] Behar O. Golden J. A. Mashimo H. Schoen F. J. Fishman M. C. (1996). Semaphorin III is needed for normal patterning and growth of nerves, bones and heart. *Nature* 383 525–528. 10.1038/383525a0 8849723

[B3] Bennett C. N. Longo K. A. Wright W. S. Suva L. J. Lane T. F. Hankenson K. D. (2005). Regulation of osteoblastogenesis and bone mass by Wnt10b. *Proc. Natl. Acad. Sci. U.S.A.* 102 3324–3329. 10.1073/pnas.0408742102 15728361 PMC552924

[B4] Bennett C. N. Ouyang H. Ma Y. L. Zeng Q. Gerin I. Sousa K. M. (2007). Wnt10b increases postnatal bone formation by enhancing osteoblast differentiation. *J. Bone Miner. Res.* 22 1924–1932. 10.1359/jbmr.070810 17708715

[B5] Brunkow M. E. Gardner J. C. Van Ness J. Paeper B. W. Kovacevich B. R. Proll S. (2001). Bone dysplasia sclerosteosis results from loss of the SOST gene product, a novel cystine knot-containing protein. *Am. J. Hum. Genet.* 68 577–589. 10.1086/318811 11179006 PMC1274471

[B6] Day T. F. Guo X. Garrett-Beal L. Yang Y. (2005). Wnt/beta-catenin signaling in mesenchymal progenitors controls osteoblast and chondrocyte differentiation during vertebrate skeletogenesis. *Dev. Cell* 8 739–750. 10.1016/j.devcel.2005.03.016 15866164

[B7] Epple H. Cremasco V. Zhang K. Mao D. Longmore G. D. Faccio R. (2008). Phospholipase Cγ2 modulates integrin signaling in the osteoclast by affecting the localization and activation of Src kinase. *Mol. Cell Biol.* 28 3610–3622. 10.1128/MCB.00259-08 18378693 PMC2423304

[B8] Faccio R. Cremasco V. (2010). PLCgamma2: where bone and immune cells find their common ground. *Ann. N. Y. Acad. Sci.* 1192 124–130. 10.1111/j.1749-6632.2009.05217.x 20392227

[B9] Fukuda T. Takeda S. Xu R. Ochi H. Sunamura S. Sato T. (2013). Sema3A regulates bone-mass accrual through sensory innervations. *Nature* 497 490–493. 23644455 10.1038/nature12115

[B10] Goetzl E. J. Lee H. Dolezalova H. Kalli K. R. Conover C. A. Hu Y. L. (2000). Mechanisms of lysolipid phosphate effects on cellular survival and proliferation. *Ann. N. Y. Acad. Sci.* 905 177–187. 10.1111/j.1749-6632.2000.tb06549.x 10818453

[B11] Gomez C. Burt-Pichat B. Mallein-Gerin F. Merle B. Delmas P. D. Skerry T. M. (2005). Expression of semaphorin-3A and its receptors in endochondral ossification: potential role in skeletal development and innervation. *Dev. Dyn.* 234 393–403. 10.1002/dvdy.20512 16145665

[B12] Goshima Y. Sasaki Y. Yamashita N. Nakamura F. (2012). Class 3 semaphorins as a therapeutic target. *Expert Opin. Ther. Targets* 16 933–944. 10.1517/14728222.2012.710201 22834859

[B13] Gu C. Rodriguez E. R. Reimert D. V. Shu T. Fritzsch B. Richards L. J. (2003). Neuropilin-1 conveys semaphorin and VEGF signaling during neural and cardiovascular development. *Dev. Cell* 5 45–57. 10.1016/S1534-5807(03)00169-2 12852851 PMC3918747

[B14] Hayashi M. Nakashima T. Taniguchi M. Kodama T. Kumanogoh A. Takayanagi H. (2012). Osteoprotection by semaphorin3A. *Nature* 485 69–76. 10.1038/nature11000 22522930

[B15] Hens J. R. Wilson K. M. Dann P. Chen X. Horowitz M. C. Wysolmerski J. J. (2005). TOPGAL mice show that the canonical Wnt signaling pathway isactive during bone development and growth and is activated by mechanical loadingin vitro. *J. Bone Miner. Res.* 20 1103–1113. 10.1359/JBMR.050210 15940363

[B16] Hsieh C. J. Kuo P. L. Hou M. F. Hung J. Y. Chang F. R. Hsu Y. C. (2015). Wedelolactone inhibits breast cancer-induced osteoclastogenesis by decreasing Akt/mTOR signaling. *Int. J. Oncol.* 46 555–562. 10.3892/ijo.2014.2769 25421824

[B17] Jaiswal N. Haynesworth S. E. Caplan A. I. Bruder S. P. (1997). Osteogenic differentiation of purified culture-expanded human mesenchymal stem cells in vitro. *J. Cell Biochem.* 64 295–312. 10.1002/(SICI)1097-4644(199702)64:2<295::AID-JCB12>3.3.CO;2-6 9027589

[B18] Kaneko S. Iwanami A. Nakamura M. Kishino A. Kikuchi K. Shibata S. (2006). A selective Sema3A inhibitor enhances regenerative responses and functional recovery of the injured spinal cord. *Nature Med.* 12 1380–1389. 10.1038/nm1505 17099709

[B19] Kikuchi K. Kishino A. Konish O. Kumagai K. Hosotani N. Saji I. (2003). In vitro and in vivo characterization of a novel semaphorin 3A inhibitor, SM 216289 or xanthofulvin. *J. Biol. Chem.* 278 42985–42991. 10.1074/jbc.M302395200 12933805

[B20] Krebsbach P. H. Kuznetsov S. A. Bianco P. Robev P. G. (1999). Bone marrow stromal cells: characterization and clinical application. *Crit. Rev. Oral Biol. Med.* 10 165–181. 10.1177/10454411990100020401 10759420

[B21] Liu Y. Q. Zhan L. B. Liu T. G. Cheng M. C. Liu X. Y. Xiao H. B. (2014). Inhibitory effect of ecliptaeherba extract and its components wedelolactone on pre-osteoclastic proliferation and differentiation. *J. Ethnopharmacol.* 157 206–211. 10.1016/j.jep.2014.09.033 25267578

[B22] Mao D. Epple H. Uthgenannt B. Novack D. V. Faccio R. (2006). PLCγ2 regulates osteoclastogenesis via its interaction with ITAM proteins and GAB2. *J. Clin. Invest.* 116 2869–2879. 10.1172/JCI28775 17053833 PMC1616195

[B23] Martin T. J. Sims N. (2005). Osteoclast-derived activity in the coupling of bone formation to resorption. *Trends Mol. Med.* 11 76–81. 10.1016/j.molmed.2004.12.004 15694870

[B24] Melo P. A. Ownby C. L. (1999). Ability of wedelolactone, heparin and p-bromophenacyl bromide to antagonized the myotosic effects of two crotaline venoms and their PLA2 myotoxins. *Toxicon* 37 199–215. 10.1016/S0041-0101(98)00183-4 9920492

[B25] Narazaki M. Tosato G. (2006). Ligand-induced internalization selects use of common receptor neuropilin-1 by VEGF165 and semaphorin3A. *Blood* 107 3892–3901. 10.1182/blood-2005-10-4113 16424390 PMC1895286

[B26] Negishi-Koga T. Takayanagi H. (2012). Bone cell communication factors and Semaphorins. *Bonekey Rep.* 1 183 10.1038/bonekey.2012.183 PMC3810552 24171101

[B27] Pederson L. Ruan M. Westendorf J. J. Khosla S. Oursler M. J. (2009). Regulation of bone formation by osteoclasts involves Wnt/BMP signaling and the chemokine sphingosine-1-phosphate. *Proc. Natl. Acad. Sci. U.S.A.* 105 20764–20769. 10.1073/pnas.0805133106 PMC2603259 19075223

[B28] Sarveswaran S. Gautam S. C. Ghosh J. (2012). Wedelolactone, a medicinal plant-derived coumestan, induces caspase-dependent apoptosis in prostate cancer cells via downregulation of PKC𝜀 without inhibiting Akt. *Int. J. Oncol.* 41 2191–2199. 10.3892/ijo.2012.1664 23076676 PMC3541032

[B29] Syed S. D. Deepak M. Yogisha S. Chandrashekar A. P. Muddarachappa K. A. D’Souza P. (2003). Trypsin inhibitory effect of wedelolactone and demethylwedelolactone. *Phytother. Res.* 17 420–421. 10.1002/ptr.1153 12722155

[B30] Takahashi T. Strittmatter S. M. (2001). Plexina1 auto inhibition by the plexin sema domain. *Neuron* 29 429–439. 10.1016/S0896-6273(01)00216-1 11239433

[B31] Takegahara N. Takamatsu H. Toyofuku T. Tsujimura T. Okuno T. Yukawa K. (2006). Plexin-A1 and its interaction with DAP12 in immune responses and bone homeostasis. *Nature Cell Biol.* 8 615–622. 16715077 10.1038/ncb1416

[B32] Teitelbaum S. L. Ross F. P. (2003). Genetic regulation of osteoclast development and function. *Nat. Rev. Genet.* 4 638–649. 10.1038/nrg1122 12897775

[B33] Wagner H. Fessler B. (1986). In vitro 5-lipoxygenase inhibition by *Eclipta* alba extracts and the coumestan derivative wedelolactone. *Planta Med.* 52 374–377. 10.1055/s-2007-969189 3797501

[B34] Worzfeld T. Offermanns S. (2014). Semaphorins and plexins as therapeutic targets. *Nat. Rev. Drug Discov.* 13 603–621. 10.1038/nrd4337 25082288

[B35] Zhang J. F. Li G. Meng C. L. Dong Q. Chan C. Y. He M. L. (2009). Total flavonoids of herba epimedii improve osteogenesis and inhibits osteoclastogenesis of human mesenchymal stem cells. *Phytomedicine* 16 521–529. 10.1016/j.phymed.2009.01.003 19394806

[B36] Zhang Z. G. Bai D. Liu M. J. Li Y. Pan J. H. Liu H. (2013). Therapeutic effect of aqueous extract from Ecliptae herba on bone metabolism of ovariectomized rats. *Menopause* 20 232–240. 10.1097/gme.0b013e318265e7dd 23096243

